# A high performance open-source syringe extruder optimized for extrusion and retraction during FRESH 3D bioprinting

**DOI:** 10.1016/j.ohx.2020.e00170

**Published:** 2021-01-01

**Authors:** Joshua W. Tashman, Daniel J. Shiwarski, Adam W. Feinberg

**Affiliations:** a Department of Biomedical Engineering, Carnegie Mellon University, Pittsburgh, PA, United States; b Department of Materials Science & Engineering, Carnegie Mellon University, Pittsburgh, PA, United States

**Keywords:** Open source, Tissue engineering, Embedded printing, Extrusion, Bioink, 3d printing, FRESH

## Abstract

Recent advances in embedded 3D bioprinting have significantly improved the resolution of individual filaments to below 100 µm; however, printing with such small filaments requires accurate extrusion of nanoliter volumes of bioink. Commercially available bioprinters and extruders are expensive and most utilize pneumatic control, which limits the minimum extrusion volume and prevents retraction (pulling bioink back into the reservoir), which is essential to printing high resolution features and complex internal geometry. Here we present a new generation of our open-source syringe pump designed for extrusion-based 3D bioprinting of soft materials: the Replistruder 4. The Replistruder 4 takes advantage of the geometry customizability and ease of 3D plastic printing while improving performance by integrating mass produced high-precision linear motion components. Simultaneously this new syringe pump remains compact and lightweight enough for several to be utilized on a 3D bioprinter for multimaterial bioprinting. To facilitate multiple use cases the Replistruder 4 is compatible with a range of syringes including disposable BD and Hamilton gastight syringes. In addition, we describe the process of designing clamps for other syringes. We demonstrate the performance of a Replistruder 4 with a 2.5 mL Hamilton gastight syringe by printing collagen type I constructs with individual filaments comprising 3.35 nL and patent channels down to 300 µm in width. With smaller volume Hamilton gastight syringes this performance can be further improved. Thus, the Replistruder 4 provides an open-source solution to print soft materials at the resolution limits of current embedded bioprinting platforms.

## Graphical abstract

**Figure:**
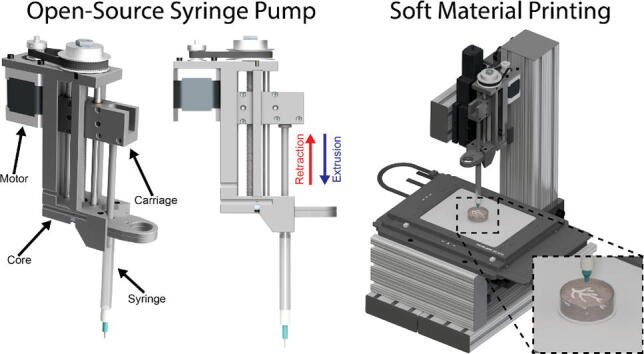

**Specifications table** [please fill in right-hand column of the table below]

**Table:** 

Hardware name	*Replistruder 4*
Subject area	• Engineering and Material Science
Hardware type	• Mechanical engineering and materials science
Open Source License	CC-BY-SA 4.0
Cost of Hardware	*$150*
Source File Repository	*https://doi.org/10.5281/zenodo.4119127*

## 1 Hardware in context

### 1.1 Introduction

The extruder is a key component of any extrusion-based 3D printing system and its performance dictates the types materials that can be used and the resolution that can be achieved. Commercial extrusion-based 3D bioprinters that cost from $5,000-$120,000 (Cellink, Allevi, EnvisionTEC) incorporate their own custom extruder designs. However, these are typically pneumatic-based for simplicity, which comes at the expense of precision and an inability to retract material (pulling back into the syringe), which is essential for controlling overextrusion during high-precision printing where other approaches such as path planning are insufficient. Likewise, high performance pneumatic extrusion systems such as the Nordson Ultimus V (~$10,000), can only prevent leakage with low viscosity inks and dispense using timed control of a constant flow rate instead of direct displacement extrusion of specific volumes [1]. We have found that a better extrusion system is a direct drive, fixed displacement pump, such as a syringe pump. By driving the plunger of a syringe forward and backward in small, precise movements, small volumes of ink can be extruded and retracted. Here we set out to design a low cost, high performance, open-source syringe pump extruder designed specifically for use with soft material 3D printers.

Our lab has previously developed a range of open-source 3D printer hardware, with a focus on syringe pump extruders for 3D bioprinting [2]. These are all purpose built for the Freeform Reversible Embedding of Suspended Hydrogels (FRESH) 3D printing technique, which extrudes hydrogels within a sacrificial support bath [3]. Our previous generation syringe pump extruder, the Replistruder 3, was designed to be almost fully 3D printed with minimal additional hardware (https://www.youmagine.com/designs/replistruder-v3-0). However, our recent version 2.0 update to the FRESH technique has dramatically improved overall print quality and required new extrusion hardware to achieve the maximum resolution and fidelity of our printed constructs [4]. To this end we developed the Replistruder 4. The goals were to retain the low cost and adaptability (e.g. mounts for new syringes) that 3D plastic printing provided in the Replistruder 3, while improving the extrusion and retraction accuracy, maximum plunger force, and ease of use by incorporating precision metal components. To do this, we 3D printed the backbone of the Replistruder 4 and mounted low-cost, mass-produced precision components to it to achieve improved rigidity and strength. The result is a <$150 syringe pump extruder designed to fulfill the requirements of high fidelity deposition of liquid and flowable materials.

### 1.2 Cost

There are a number of 3D printer extruders currently available. Commercial bioprinters, with their own custom extrusion tools, cost from $5,000-$120,000 (e.g, Cellink, Allevi, EnvisionTEC). Standalone precision pneumatic extruders, such as the Nordson Ultimus V cost about $10,000. As mentioned previously, these pneumatic extruders are used widely for 3D printing but have difficulty controlling extrusion and retraction. As an alternative, several groups, including our own, have designed and released their own syringe pump extruders to drive down price and increase accessibility of 3D bioprinting research. Most of these pumps rely heavily on 3D printed parts, which reduces cost, but can limit precision. These designs are also often too large to allow for compact, multi-material printheads [5], [6], [7]. Improvements in the FRESH embedded printing technique have enabled printing at very high resolution (20 µm filament diameters) that exceeds the capabilities of these current designs.

The Replistruder 4 described here combines 3D printed parts with precision, low cost linear motion components to construct a compact, high-performance syringe pump for $141 (see *Bill of Materials*). A range of commonly used syringes from ~10 mL down to 1 µL (we have included designs for 2.5 mL, 5 mL, and 10 mL syringes) can be loaded into the Replistruder 4 and it can be easily mounted on standard desktop thermoplastic 3D printers (e.g. Lulzbot Mini 2, Makergear M2, Flashforge Creator Pro). All of the plastic parts are printed using a Prusa i3 MK3 desktop 3D printer and the remaining components such as the steel rods, nuts, bolts, and belts are purchased from online retailers (e.g. McMaster Carr, Amazon).

### 1.3 Extrusion based soft material printing

In the field of tissue engineering there is considerable interest in printing soft materials. Traditional 3D bioprinting has been challenging due to the inability of biological hydrogels to support their own weight in air during printing. With recent advances in embedded 3D printing approaches, such as FRESH, the materials are supported during the printing process, but improved extrusion capability is necessary [8]. A range of requirements exist for printing. Some printing applications require large volumes (creating a large tissue phantom such as a human scale heart), while others require minute volumes (printing with ultra-high cell concentrations) [4], [9]. The pressures necessary to extrude these materials also vary widely. As such, to be generally applicable an open-source syringe pump must be able to extrude and retract accurately across a range of materials and volumes. A list of performance requirements can help define the steps necessary to achieve this:

1. A rigid core must be constructed as a foundation to prevent deflection during the application of force on the syringe plunger.
2. Precision linear motion components must be implemented around this core to produce accurate, repeatable translation and positioning of a moving carriage (for depressing the syringe plunger).
3. To drive the linear translation a high quality, 0.9-degree stepper motor can be used in combination with gearing to drive down the minimum translation distance (and therefore minimal extrusion volume).
4. The syringe itself must be exchangeable to adjust total printable volume, as well as minimal extrusion volume.
5. The entire assembly needs to be light enough and compact enough for multiple pumps to fit on printers without affecting their motion performance.

### 1.4 Replistruder 4 performance

The Replistruder 4 is a syringe pump extruder capable of extruding and retracting small volumes, which can be calculated theoretically from some simple calculations. The drivetrain, which utilizes a 400 step per rotation NEMA 17 stepper motor, requires 960 full steps to move the syringe plunger 1 mm. This means each full step (with a step angle accurate to ± 5%) will lead to a linear translation of 1.04 ± 0.052 µm. If a Hamilton 1000 series 2.5 mL Gastight syringe is used, 1.04 µm corresponds to a swept volume of the plunger of 43.4 ± 2.17 nL. If we take 1/16 microstepping into account, which is common in 3D printing hardware, then the number of steps required to extrude 1 mm is 15,360 and the swept volume of a microstep is 2.71 ± 0.14 nL. These volumes seem extremely small, but they must be taken in context of the objects we intend to print with the hardware. In our lab, though we have printed filaments down to 20 µm using pulled glass pipette tips, we more often use 100 µm diameter needles on the Replistruder 4. If we expect to print a 1 mm long filament of collagen with this needle at a 50 µm layer height, then we must be able to precisely extrude 5 nL (approximating the volume as a rectangular prism). We have routinely printed such small features using the Replistruder 4 (as shown in the validation and characterization section). Further improvements in resolution are capable with increased microstepping, but it is preferred to utilize hardware modifications instead, in the form of a smaller volume syringe. For example, with the Hamilton 1710 100 µl syringe (a useful total print volume for small constructs), the swept volume of a microstep drops to 109 ± 5.5 pL.

While the overall print performance depends strongly on the rigidity and dynamics of the print platform, we have had good results bioprinting with the Replistruder 4 mounted to modified commercial plastic printers. On these platforms, which are typically less rigid, we use printing speeds ranging from 10 to 20 mm/s and travel speeds ranging from 20 to 30 mm/s. Without improvements to the printing platform an increase in speed typically results in lower quality minimum printable feature sizes, but larger prints without intricate internal void features can successfully be printed at the upper limits of these speed ranges with high fidelity.

The Hamilton gastight syringes provide additional performance benefits beyond their high accuracy control of fluids. As they are built from easily cleaned materials (e.g. stainless steel, Teflon, glass) and can utilize inexpensive dispense tips that are reusable and compatible with a wide range of bioinks and even synthetics such as silicone. Furthermore, they can be autoclaved to ensure sterility for delicate cellularized prints or acellular scaffolds for animal experiments.

## 2 Hardware description

**Fig. 1:**
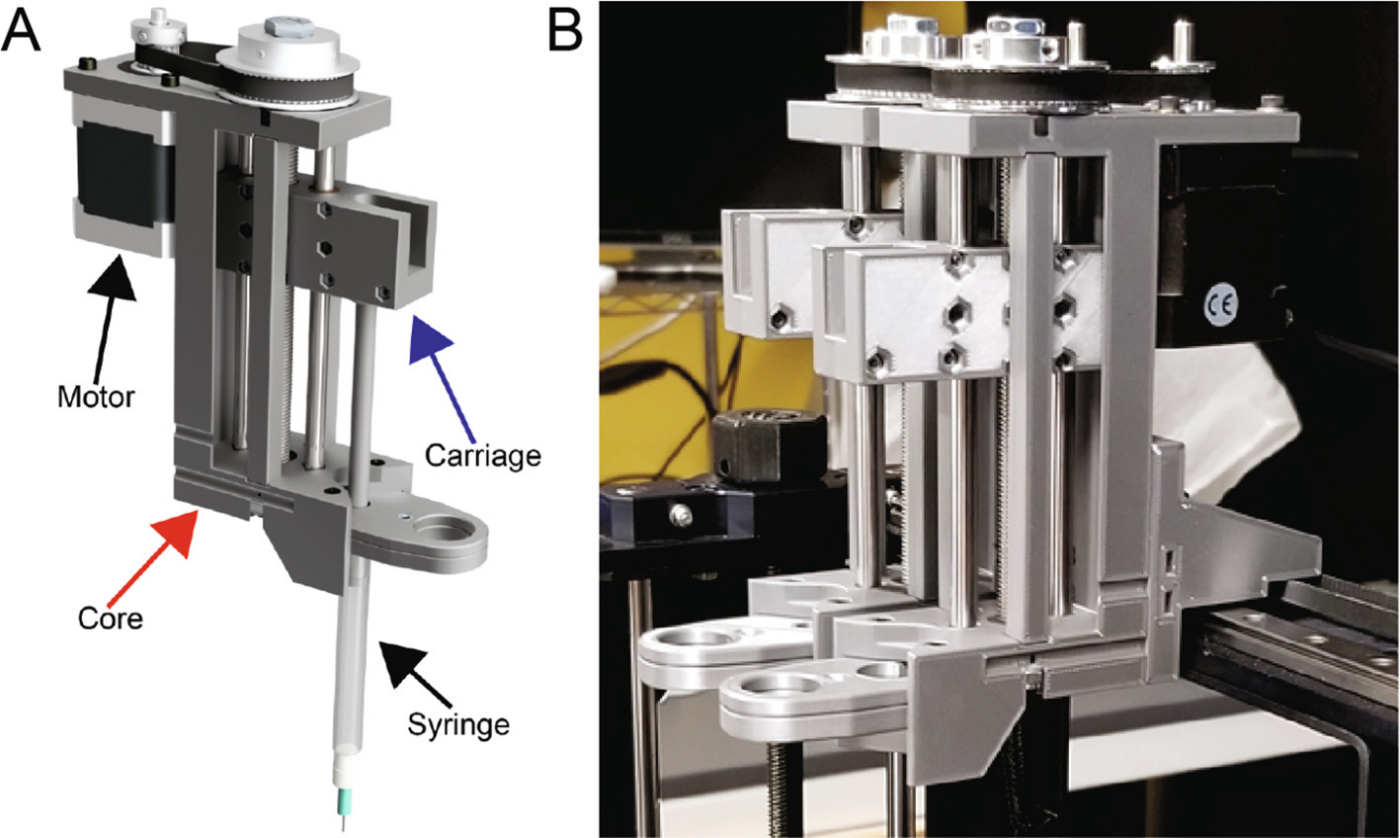
**Replistruder 4. A)** An isometric view render of the Replistruder 4 with the core highlighted by a red arrow and the carriage highlighted with a blue arrow. **B)** Two Replistruder 4 syringe pumps mounted on a bioprinter for dual material printing. (For interpretation of the references to colour in this figure legend, the reader is referred to the web version of this article.)

The Replistruder 4 consists of two main assemblies shown in Fig. 1A: [1] the core (red arrow), which provides a rigid backbone for the linear motion components and a mounting point for the syringe, and [2] the carriage (blue arrow). As the screw turns the carriage is linearly translated with guidance from the outer core structure. A syringe is mounted to the outer core structure and as the carriage moves it depresses the plunger of the syringe (to which it is rigidly coupled with a bolt). In this way the linear motion of the carriage results in extrusion or retraction of material from the syringe depending on the direction of travel. The volume and rate of extrusion or retraction are determined by the distance the carriage moves and the speed at which it moves. The accuracy and performance of this type of design depends on the rigidity of the syringe pump, the precision of its components and motion control, and the syringe specifications. The goal of the Replistruder 4 was to increase its performance over mostly plastic syringe pump extruders by using standard precision metal components while limiting the increase in cost to allow for widespread adoption. As a result, the Replistruder 4 is a precise syringe pump extruder that can be fabricated and assembled by even novice users. All designs are licensed as open source under a CC BY-SA 4.0 license. The non-printed components consist of standard hardware such as bearings, linear motion rods, nuts and bolts, 3D printer belts, stepper motors, and syringes.

### 2.1 Core

The core (Fig. 1A) is the backbone of the Replistruder 4 and provides mounting points for the linear motion hardware, the motor, the syringe, and for attachment to a 3D printer carriage. The design of the extruder core takes advantage of the capability of 3D plastic printing to produce geometries that would be difficult and expensive to produce with traditional machining or casting approaches and has been optimized to not require supports during the printing process. Two precision ground case hardened linear motion shafts are supported on their ends by slip-fit holes in the core, these guide the linear motion of the carriage. The leadscrew, which moves the carriage, is supported on both ends by oil-impregnated thrust supporting sleeve bearings that sit within press fit pockets that align the screw between the two linear motion shafts. The motor mounts directly to the core and is coupled to the leadscrew by a GT2 belt and pulley system (which rides on the oil impregnated thrust bearings) with a 3:1 gearing ratio. The mounting points for the motor are slotted to allow for tensioning of the belt. The syringe itself is held in an easy to transport, customizable flange clamp that slots into the core and is mounted with M3 hardware that provides redundant clamping. The v shaped profile of the extruder mount allows for rigid support of the syringe to minimize deflection during printing.

### 2.2 Carriage

**Fig. 2:**
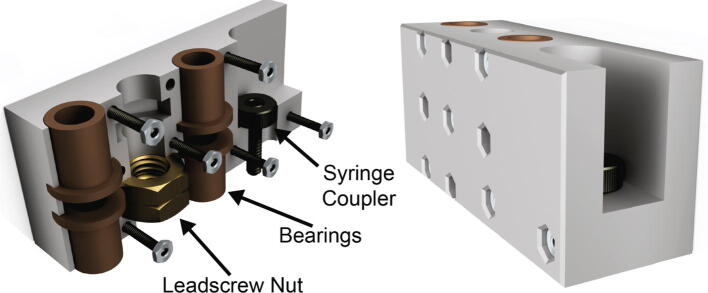
**Extruder Carriage.** The extruder carriage is composed of two halves that trap bearings and the leadscrew nut when they are bolted together. The front of the carriage bolts to the head of the syringe.

The carriage (Fig. 1A and 2) translates the linear motion provided by the core into depression of the syringe plunger, which leads to extrusion of material. The two mirror halves of the carriage capture four ultra-low friction Teflon oil impregnated flanged sleeve bearings, two for each linear motion shaft. The redundant bearings improve rigidity and allow the carriage to support a large moment while still moving smoothly and precisely. This in turn allows the Replistruder 4 to apply significant force to the syringe plunger without deflection of the carriage assembly. To couple the carriage to the leadscrew two brass hex nuts are captured within a hexagonal pocket that applies sufficient preload to the nuts to significantly reduce backlash while still allowing the motor to turn the leadscrew rapidly and precisely. Finally, the carriage couples to the syringe with either 6–32 or M3 hardware (depending on the syringe), allowing for both positive and negative displacement of the plunger, which is essential for high precision printing. (Fig. 2)

### 2.3 Key aspects of the hardware

• At a total cost of $141, the Replistruder 4 is a fully customizable, open-source, low cost alternative to commercial syringe pumps.
• Because of its compact form and high precision, the Replistruder 4 is ideal for use as an extruder on a biomaterial or synthetic soft material printer. Depending on syringe choice the Replistruder 4 weighs ~ 600 g.
• The Replistruder 4 can easily be mounted to existing printers through an adapter plate.
• The Replistruder 4 uses a NEMA 17 stepper motor, which can be driven by all 3D printing platforms and many other motion control platforms.
• The Replistruder 4 is adaptable to many different syringe types, through modification of the template syringe holder.

## 3 Design files

Design Files Summary

**Table:** 

Design File Name	Design File Type	Open Source License	Location of File
Replistruder4_Core	CAD and PDF FIle	CC-BY-SA 4.0	https://doi.org/10.5281/zenodo.4119127
Replistruder4_CoreSupport	CAD and STL FIle	CC-BY-SA 4.0	https://doi.org/10.5281/zenodo.4119127
Replistruder4_ECarriage_1	CAD and STL FIle	CC-BY-SA 4.0	https://doi.org/10.5281/zenodo.4119127
Replistruder4_ECarriage_2	CAD and STL FIle	CC-BY-SA 4.0	https://doi.org/10.5281/zenodo.4119127
Replistruder4_2_5_HamiltonBottom	CAD and STL FIle	CC-BY-SA 4.0	https://doi.org/10.5281/zenodo.4119127
Replistruder4_2_5_HamiltonTop	CAD and STL FIle	CC-BY-SA 4.0	https://doi.org/10.5281/zenodo.4119127
Replistruder4_5_HamiltonBottom	CAD and STL FIle	CC-BY-SA 4.0	https://doi.org/10.5281/zenodo.4119127
Replistruder4_5_HamiltonTop	CAD and STL FIle	CC-BY-SA 4.0	https://doi.org/10.5281/zenodo.4119127
Replistruder4_10_BDBottom	CAD and STL FIle	CC-BY-SA 4.0	https://doi.org/10.5281/zenodo.4119127
Replistruder4_10_BDTop	CAD and STL FIle	CC-BY-SA 4.0	https://doi.org/10.5281/zenodo.4119127
Mini2AdapterPlate	CAD and STL FIle	CC-BY-SA 4.0	https://doi.org/10.5281/zenodo.4119127
Mini2MountBracket	CAD and STL FIle	CC-BY-SA 4.0	https://doi.org/10.5281/zenodo.4119127

**Brief description of each part**

**Replistruder4_Core:** This 3D printed plastic part forms the backbone of the Replistruder 4 syringe pump. All of the other components are attached to this core and the core itself has mounting points for attaching the Replistruder 4 to a 3D printer, or other device.

**Replistruder4_CoreSupport:** These 3D printed plastic parts provide additional rigidity, in conjunction with M5 bolts, to the Replistruder4_Core.

**Replistruder4_ECarriage_1, 2:** These are each one half of the Replistruder 4′s extruder carriage. Together they capture the leadscrew nut and linear motion bearings that allow for linear translation of the extruder carriage.

**Replistruder4_2_5_HamiltonBottom:** This is the bottom half of a syringe mount for a 2.5 mL Hamilton Series 1000 Gastight Syringe.

**Replistruder4_2_5_HamiltonTop:** This is the top half of a syringe mount for a 2.5 mL Hamilton Series 1000 Gastight Syringe.

**Replistruder4_5_HamiltonBottom:** This is the bottom half of a syringe mount for a 5 mL Hamilton Series 1000 Gastight Syringe.

**Replistruder4_5_HamiltonTop:** This is the top half of a syringe mount for a 5 mL Hamilton Series 1000 Gastight Syringe.

**Replistruder4_10_BDBottom:** This is the top half of a syringe mount for a 10 mL BD plastic syringe.

**Replistruder4_10_BDTop:** This is the bottom half of a syringe mount for a 10 mL BD plastic syringe.

**Mini2MountBracket:** This is an example of a mount for the Replistruder 4 that has been used to attach it to a Lulzbot Mini 2 adapted to bioprinting.

**Mini2AdapterPlate:** This adapts the bolt pattern on the back of the Replistruder 4 to the bolt pattern of the Min2MountBracket

## 4 Bill of materials

**Table:** 

Designator	Component	Number of Units	Cost Per Unit [USD]	Total Cost [USD]	Source of Materials	Material Type
M8 × 1.25 mm, 150 mm long, Fully Threaded Socket Cap	B01MYM6J0C	1	2.67	2.67	Amazon	Stainless Steel
M8 × 1.25 mm, Brass Thin Hex Nut	93187A300	2	0.41	0.82	McMaster	Brass
Flanged, Oil-Embedded Sleeve Bearing for ¼” Shaft	1677 K3	4	1.11	4.44	McMaster	Bronze
M2 × 0.4 mm, 20 mm long, Fully Threaded Socket Cap	91290A049	6	0.13	0.78	McMaster	Steel
M2 × 0.4 mm, Medium-Strength Steel Thin Hex Nut	90695A025	6	0.041	0.25	McMaster	Steel
M5 × 0.8 mm, 130 mm long, Partially Threaded Socket Cap	91290A141	2	1.80	3.6	McMaster	Steel
M5 × 0.8 mm, Steel Nylon-Insert Locknut	90576A104	2	0.05	0.10	McMaster	Steel
¼” Case Hardened Linear Shaft	6061 K413	2	5.18	10.36	McMaster	Steel
Flanged, Oil-Embedded Sleeve Bearing for 8 mm Shaft	6659 K677	2	2.35	4.70	McMaster	Bronze
60-Tooth 8 mm Bore GT2 Pulley	B01G1N1CI8	1	4.50	4.50	Amazon	Aluminum
0.9 Degree Stepper Motor	High Torque Axis Motor	1	24.99	24.99	Filastruder	Electronics
180 mm, 6 mm wide GT2 Belt	B014QLQBQ0	1	1.40	1.40	Amazon	Rubber
20-Tooth 5 mm Bore GT2 Pulley	B07MN2C5WJ	1	2.19	2.19	Amazon	Aluminum
M8 × 1.25 mm, Steel Thin Hex Nut	90370A101	1	0.2	0.2	McMaster	Steel
M3 × 0.5 mm, 10 mm long, Fully Threaded Socket Cap	91290A115	4	0.08	0.32	McMaster	Steel
M3 × 0.5 mm, Steel Thin Hex Nut	90695A033	5	0.04	0.20	McMaster	Steel
M3 × 0.5 mm, 6 mm long, Fully Threaded Socket Cap	91290A111	3	0.09	0.27	McMaster	Steel
2.5 mL Hamilton Gastight Model 1002	81,420	1	54.00	54.00	Hamilton	Multiple
M3 × 0.5 mm, 20 mm long, Fully Threaded Socket Cap	91290A123	2	0.11	0.22	McMaster	Steel
6–32, 17/32″ long, Thumb Screw with Hex Drive	98704A210	1	0.6	0.6	McMaster	Steel
Black PLA	MY6CYEZM	1	19.99	19.99	Matter Hackers	PLA

## 5 Build instructions

The majority of the Replistruder 4 is 3D printed from PLA filament. All STL files have been prepared and saved in their intended printing orientation. During the printing process no supports are required. All parts are recommended to be printed with 60% infill and 3 perimeters with a layer height of 0.25 mm. Some print settings may vary depending upon the printer used.

### 5.1 Extruder carriage assembly

Begin assembly of the Replistruder 4 by building the extruder carriage. For this assembly you need:

(1x) M8 × 1.25 mm, 150 mm long, Fully Threaded Socket Cap
(2x) M8 × 1.25 mm, Brass Thin Hex Nut
(1x) Replistruder4_ECarriage_1
(1x) Replistruder4_ECarriage_2
(4x) Flanged, Oil-Embedded Sleeve Bearing for ¼” Shaft
(6x) M2 × 0.4 mm, 20 mm long, Fully Threaded Socket Cap
(6x) M2 × 0.4 mm, Medium-Strength Steel Thin Hex Nut

**Fig. 3:**
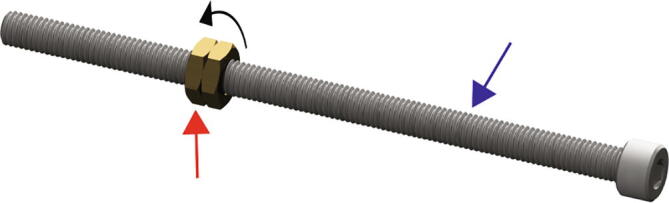
Leadscrew Nut Threading.

Thread both of the M8 brass thin hex nuts (Fig. 3 red arrow) onto the 150 mm long M8 socket cap bolt (Fig. 3 blue arrow)

**Fig. 4:**
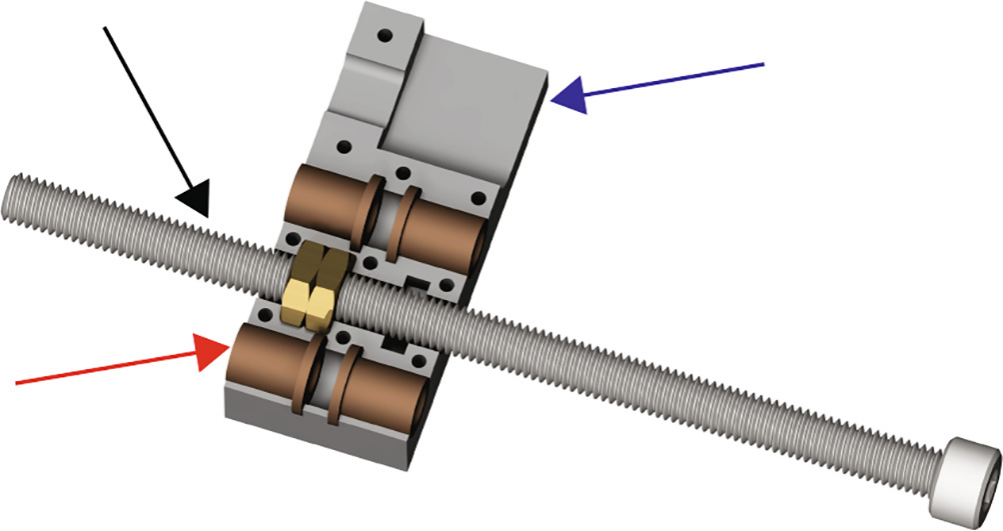
Extruder Carriage Assembly 1.

Place the threaded leadscrew nuts (Fig. 4 black arrow) into the larger hexagonal pocket found near the center of the Replistruder4_ECarriage_2 (Fig. 4 blue arrow). Make sure to align the nuts to seat well in the pocket. Next place four flanged sleeve bearings into their respective pockets (Fig. 4 red arrow).

**Fig. 5:**
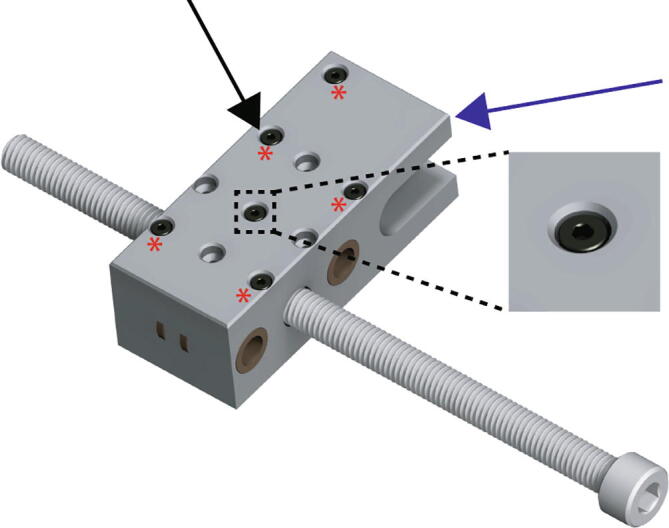
Extruder Carriage Assembly 2.

Place the Replistruder4_ECarriage_1 (Fig. 5 blue arrow), on top of the assembly, encasing the bearings and the leadscrew nuts. Insert the six M2 20 mm long bolts in the pattern shown in Fig. 5 (black arrow, zoom, red asterisks).

**Fig. 6:**
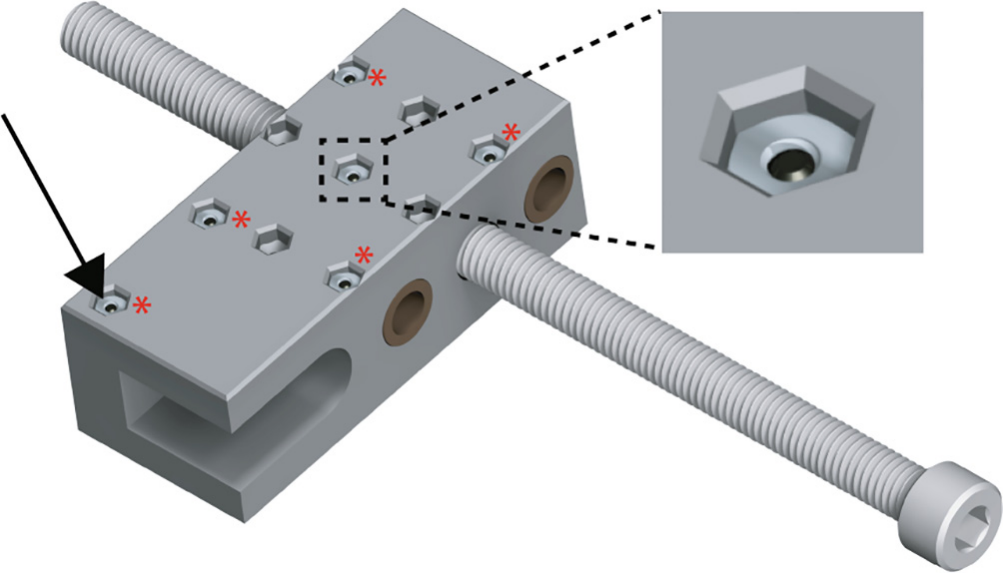
Extruder Carriage Assembly 3.

Flip the assembly over, taking care to keep the 20 mm long M2 bolts from falling out. Insert the six M2 thin nuts in the appropriate hexagonal pockets (Fig. 6 black arrow, zoom, red asterisks). Tighten the M2 bolts until snug, excessive force is not necessary.

### 5.2 Extruder core assembly

Continue assembly of the Replistruder 4 by building the main core. For this assembly you need:

(1x) Replistruder4_Core
(2x) Replistruder4_CoreSupport
(2x) M5 × 0.8 mm, 130 mm long, Partially Threaded Socket Cap
(2x) M5 × 0.8 mm, Steel Nylon-Insert Locknut

**Fig. 7:**
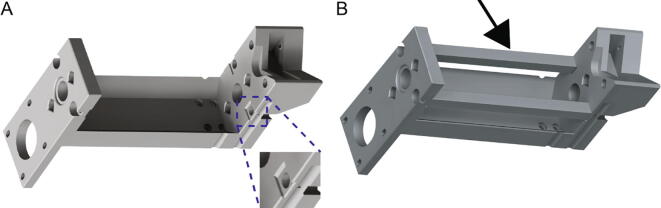
Extruder Core Assembly 1. A) Core with zoom in of pocket. B) Core with supports inserted.

Into their pockets on the sides of the Replistruder4_Core (Fig. 7 left, zoom) insert the two Replistruder4_CoreSupport (Fig. 7 right, black arrow).

**Fig. 8:**
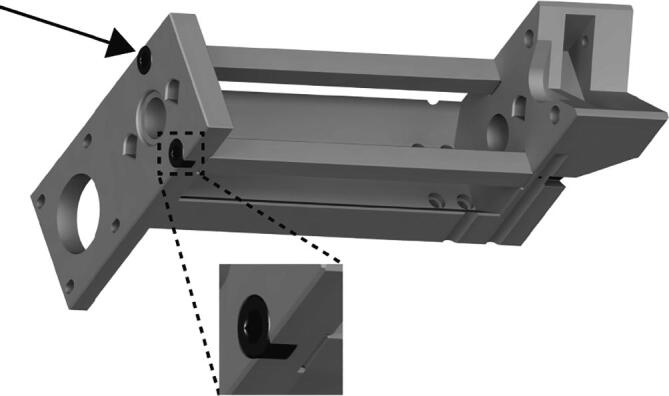
Extruder Core Assembly 2.

Insert the two 130 mm long M5 socket cap bolts (Fig. 8 black arrow, zoom) into the Replistruder4_CoreSupport pieces.

**Fig. 9:**
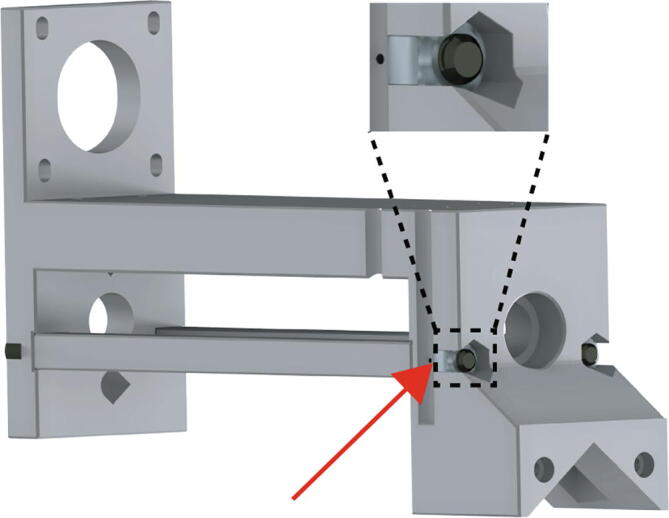
Extruder Core Assembly 3.

Flip the assembly and insert the two M5 nylon locknuts (Fig. 9 red arrow, zoom) into their hexagonal pockets in the Replistruder4_Core.Tighten until the nylon locknuts are seated.

### 5.3 Extruder carriage Mounting

Continue assembly of the Replistruder 4 by mounting the carriage to the main core. For this assembly you need:

(1x) Extruder Carriage Assembly
(1x) Extruder Core Assembly
(2x) Flanged, Oil-Embedded Sleeve Bearing for 8 mm Shaft
(2x) ¼” Case Hardened Linear Shaft
(1x) 60-Tooth 8 mm Bore GT2 Pulley
(1x) M8 × 1.25 mm, Steel Thin Hex Nut

**Fig. 10:**
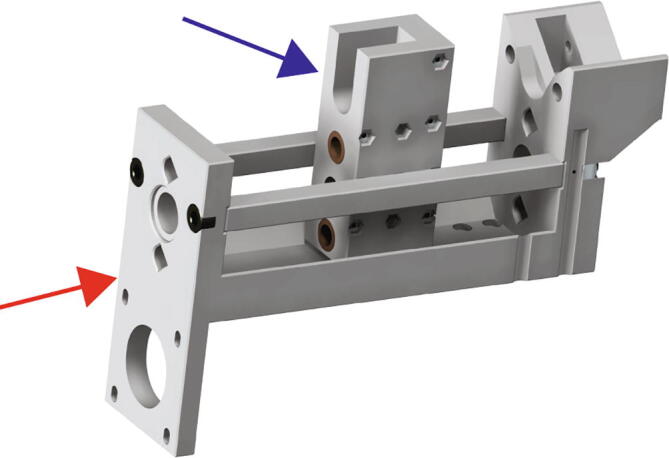
Extruder Carriage Mounting 1.

Begin by removing the 150 mm long M8 socket cap bolt from the extruder carriage assembly (Fig. 6) and positioning the assembly (Fig. 10 blue arrow) within the extruder core assembly (Fig. 10 red arrow).

**Fig. 11:**
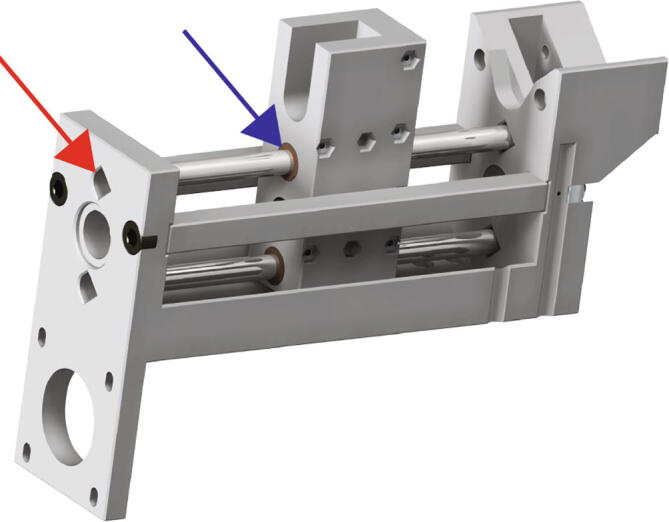
Extruder Carriage Mounting 2.

Next, insert the ¼” linear shafts through the diamond shaped holes in the extruder core assembly (Fig. 11 red arrow), through the sleeve bearings within the extruder carriage assembly (Fig. 11 blue arrow) and back into the extruder core assembly.

**Fig. 12:**
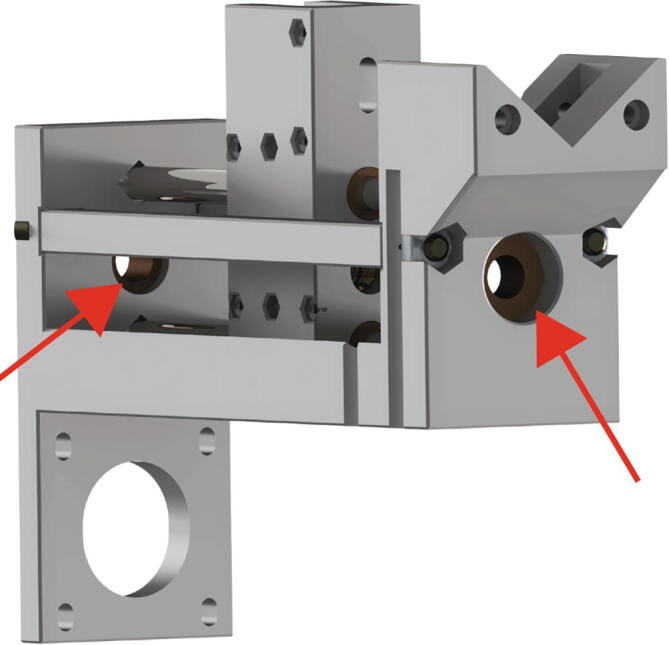
Extruder Carriage Mounting 3.

After inserting the linear shafts, insert the two flanged bearings for the 8 mm shaft in the pockets on opposite sides of the Replistruder4_Core (Fig. 12 red arrows).

**Fig. 13:**
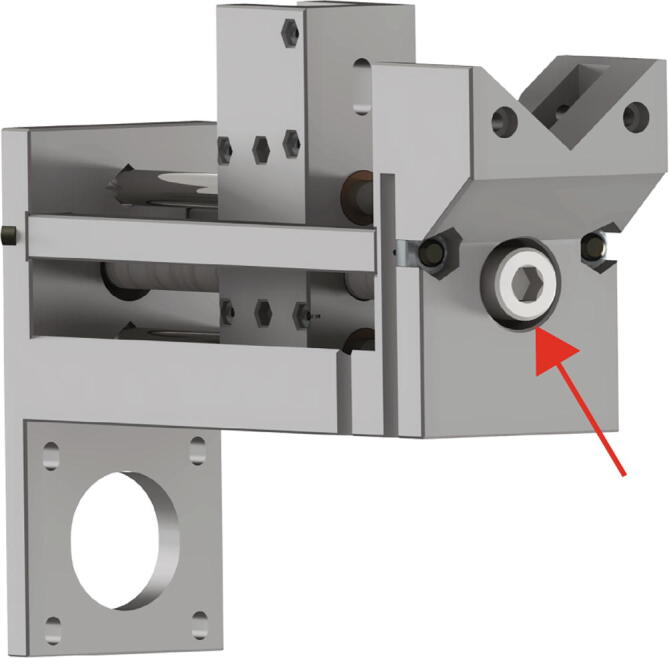
Extruder Carriage Mounting 4.

Next, insert the 150 mm long M8 socket cap bolt through the 8 mm flanged bearing at the bottom, then thread it through the extruder carriage assembly, and finally through the second 8 mm flanged bearing, as shown in Fig. 13 by the red arrow.

At this point we need to lock the screw in place on the far side, to prevent translation. To do this we use the 60-tooth GT2 pulley and the M8 steel thin nut.

**Fig. 14:**
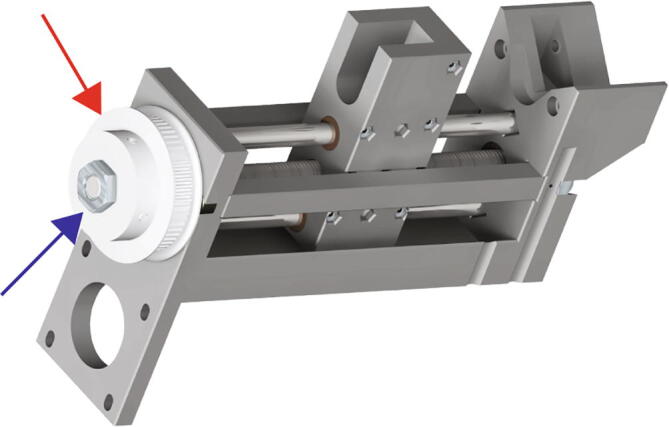
Extruder Carriage Mounting 4.

Retract the set screws in the 60 tooth GT2 pulley (Fig. 14 red arrow) and then slide it onto the M8 bolt. Next finger tighten the M8 steel thin nut (Fig. 14 blue arrow), just tight enough to prevent linear translation of the M8 bolt. Next, tighten the set screws on the 60-tooth GT2 pulley to lock the assembly in place (tighten significantly, as this determines maximum extrusion force).

### 5.4 Motor Mounting

Continue assembly of the Replistruder 4 by mounting the motor to the extruder core assembly. For this assembly you need:

(1x) Extruder Core Assembly
(1x) 0.9 Degree Stepper Motor
(1x) 20-Tooth 5 mm Bore GT2 Pulley
(1x) 180 mm, 6 mm wide GT2 Belt
(4x) M3 × 0.5 mm, 10 mm long, Fully Threaded Socket Cap

**Fig. 15:**
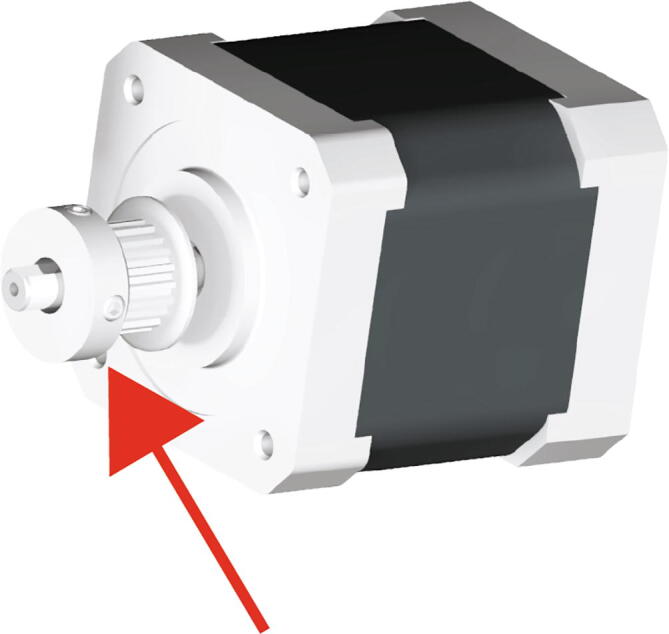
Extruder Motor Mounting 1.

Begin by mounting the 20 tooth GT2 pulley to the stepper motor as in Fig. 15 (red arrow).

**Fig. 16:**
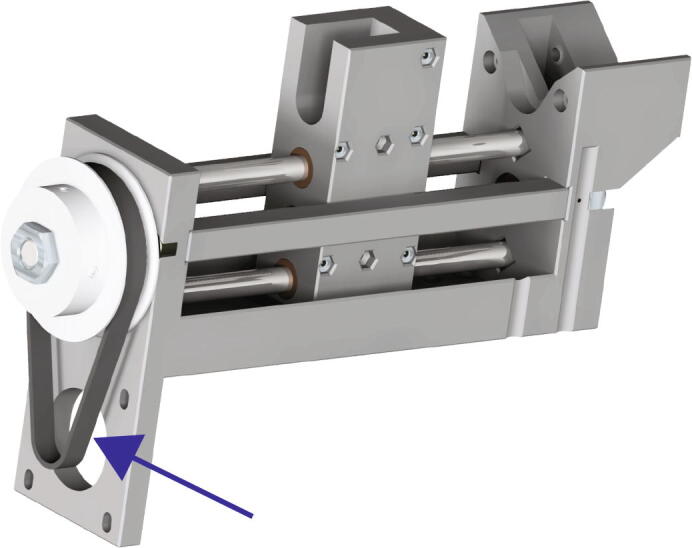
Extruder Motor Mounting 2.

Next, place the 180 mm belt around the 60-tooth pulley as in Fig. 16 (blue arrow).

**Fig. 17:**
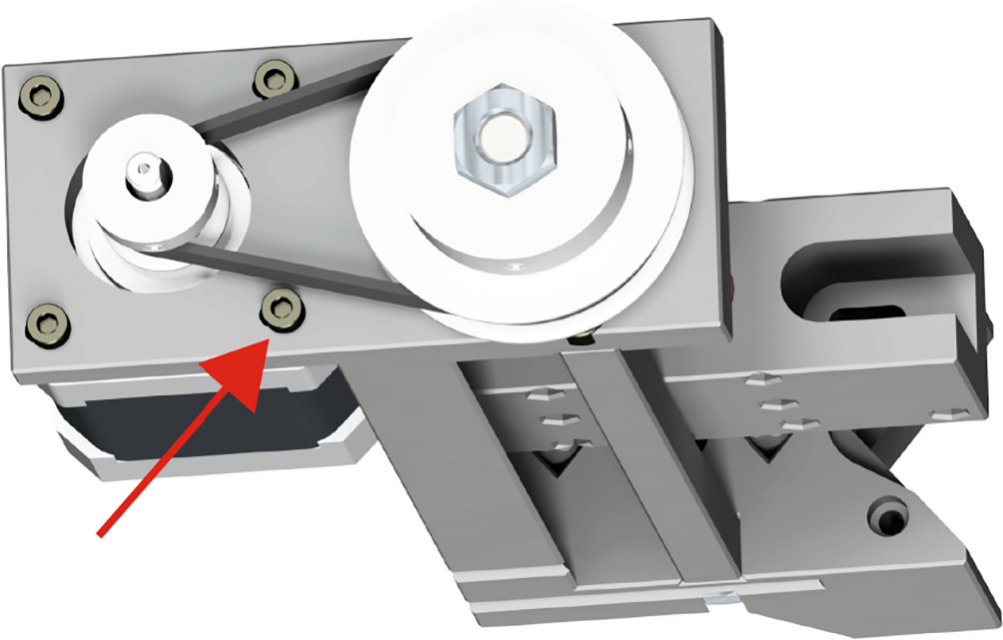
Extruder Motor Mounting 3.

**Fig. 18:**
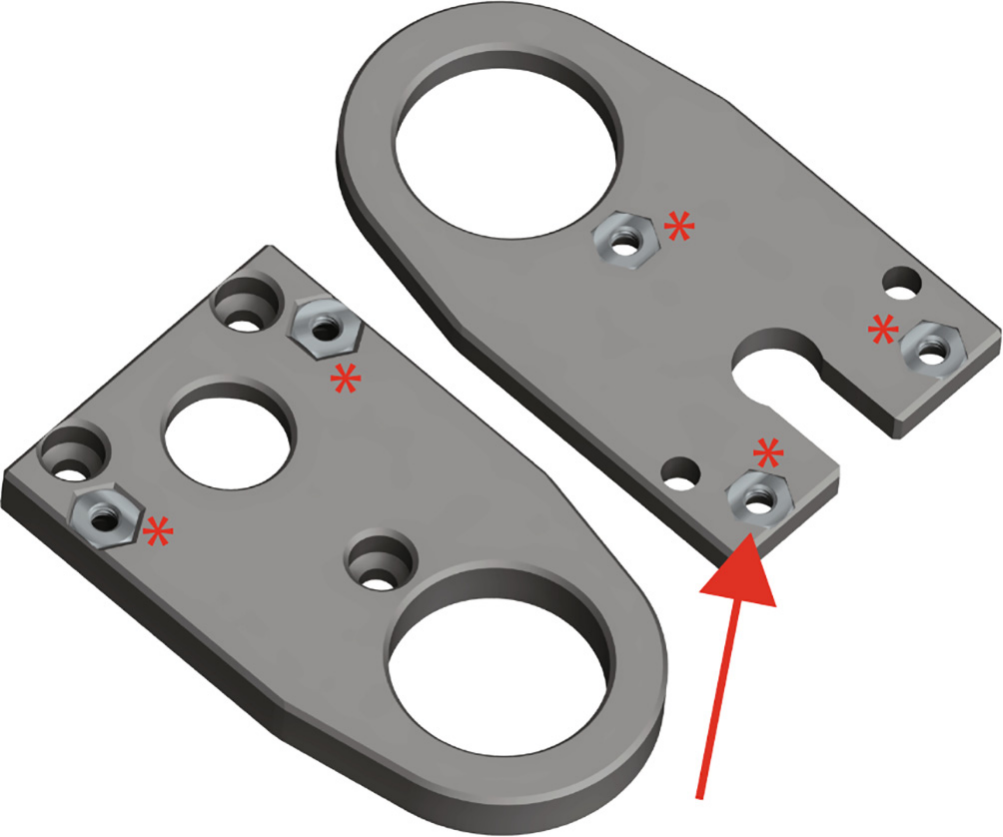
Extruder Syringe Mounting 1.

Insert the motor pulley through the belt as in Fig. 18. Screw the four 10 mm long M3 socket cap bolts into the motor (Fig. 17 red arrow). Do not tighten them completely. Ensure that the 20-tooth pulley is at the correct height, such that the belt is not pulled upwards or downwards. Once the height has been adjusted, lock the position by tightening the sets screws within the 20-tooth pulley. Next, put the belt in tension by pulling the motor away from the extruder core assembly. While the belt is taut tighten the four 10 mm long M3 bolts.

### 5.5 Extruder syringe Mounting

Continue assembly of the Replistruder 4 by mounting the syringe to the extruder assembly. For this assembly you need:

(1x) Extruder Assembly
(5x) M3 × 0.5 mm, Steel Thin Hex Nut
(1x) 2.5 mL Hamilton Gastight Model 1002
(1x) Replistruder4_2_5_HamiltonBottom
(1x) Replistruder4_2_5_HamiltonTop
(3x) M3 × 0.5 mm, 6 mm long, Fully Threaded Socket Cap
(2x) M3 × 0.5 mm, 20 mm long, Fully Threaded Socket Cap
(1x) 6–32, 17/32″ long, Thumb Screw with Hex Drive

First, insert the five M3 steel thin nuts into the corresponding hexagonal pockets in Replistruder-4_2_5_HamiltonBottom and Replistruder4_2_5_HamiltonTop (Fig. 18 red arrow)

**Fig. 19:**
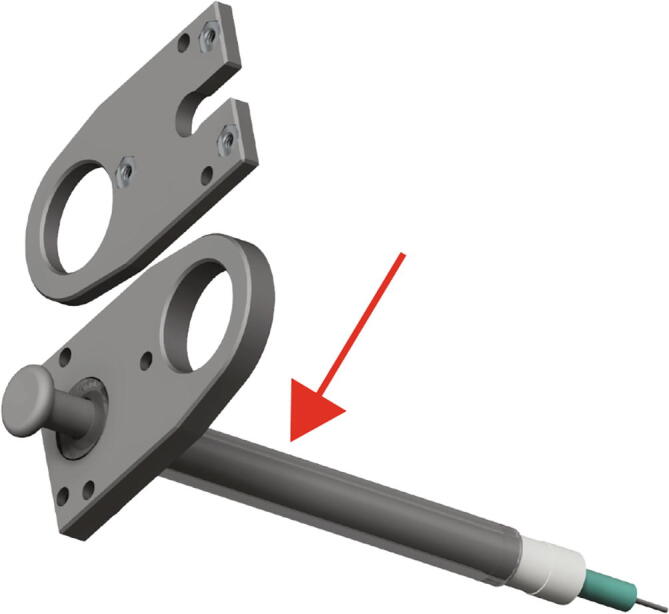
Extruder Syringe Mounting 2.

Next, insert the 2.5 mL Hamilton syringe into the Replistruder4_2_5_HamiltonBottom as in Fig. 19 (red arrow, red asterisks).

After inserting the Hamilton syringe, we need to clamp it in place. We will do this with the three 6 mm long M3 socket cap bolts.

**Fig. 20:**
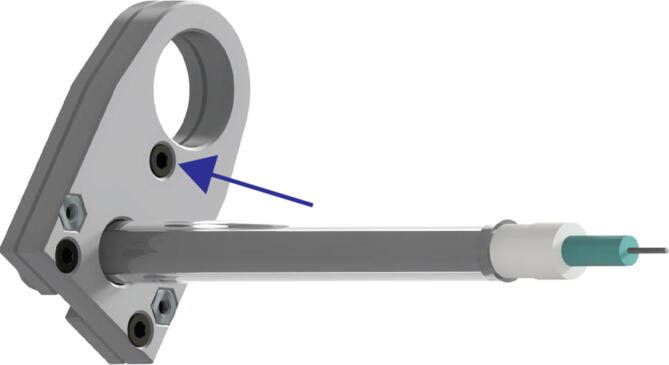
Extruder Syringe Mounting 3.

Insert the 6 mm long M3 socket cap bolts into the corresponding holes in the underside of Replistruder4_2_5_HamiltonBottom as in Fig. 20 (blue arrow, zoom).

**Fig. 21:**
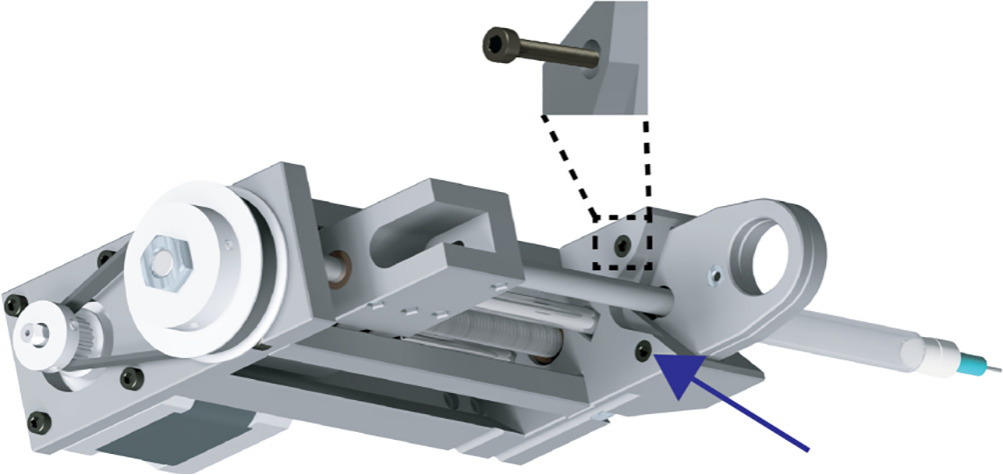
Extruder Syringe Mounting 4.

Next, slot the clamped syringe into the receptacle in the front of the extruder assembly, (make sure to retract the extruder carriage to allow for the plunger of the syringe to fit). Secure the clamped syringe to the extruder assembly using the two M3 20 mm long socket cap bolts (Fig. 21 blue arrow, zoom).

**Fig. 22:**
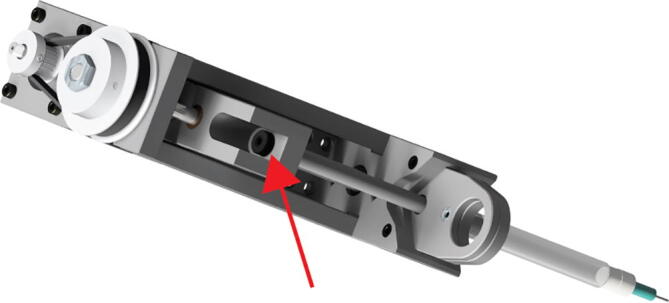
Extruder Syringe Mounting 5.

Finally, advance the extruder carriage and attach it to the syringe plunger (Hamilton syringes have a threaded plunger) using the 6–32, 17/32″ long, Thumb Screw with Hex Drive (Fig. 22 red arrow).

## 6 Operating instructions

**Fig. 23:**
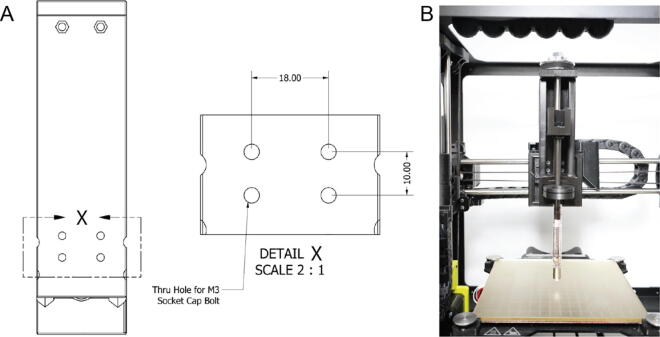
**Replistruder 4 Mounting. A)** Bolt pattern, with detail zoom X. **B)** Example mount on Lulzbot Mini 2.

To operate the Replistruder 4 it needs to be mounted on a 3D printer and loaded with a syringe. Mounting the Replistruder 4 is relatively straightforward. An adapter with a 10 mm × 18 mm rectangular M3 socket cap bolt pattern as shown in the detail X callout of Fig. 23A can be 3D printed**.** The M3 bolts will pass through the body of the Replistruder 4 and protrude on the motor side, the heads of the bolts will be recessed. An example of mounting the Replistruder 4 to a Lulzbot Mini 2 3D printer is shown in Fig. 23B (Mini2MountBracket.stl and Mini2AdapterPlate.stl for Lulzbot Mini 2 provided for download as an example).

**Fig. 24:**
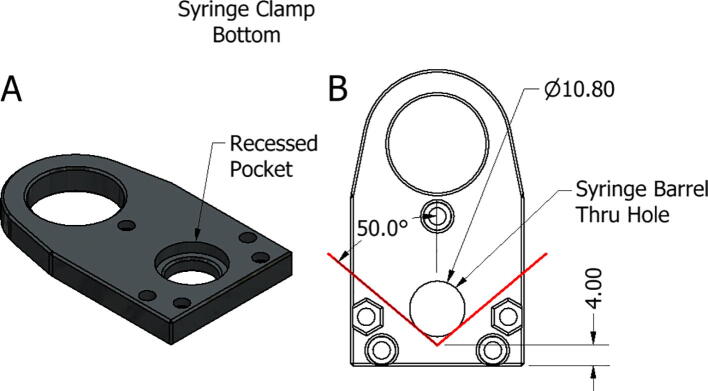
**Replistruder Syringe Clamp Bottom. A)** Isometric view of syringe clamp bottom **B)** Schematic for determining barrel thru hole location.

To customize the syringe clamps for a new syringe type, the design starts with the bottom of the syringe clamp. As can be seen in Fig. 24A this side of the clamp has a recessed pocket and a thru hole for the barrel of the syringe. In Fig. 24B the specifications for locating the syringe barrel thru hole can be found. The thru hole must be made tangent to a 100° wedge spaced 4 mm from the mounting side of the clamp. An appropriately deep pocket, concentric to the thru hole, can then be extruded to make the syringe flange flush with the top of the clamp.

**Fig. 25:**
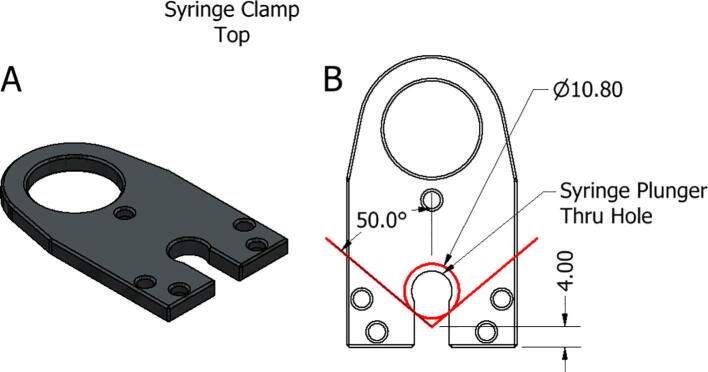
**Replistruder Syringe Clamp Top. A)** Isometric view of syringe clamp top **B)** Schematic for determining plunger thru hole location.

To customize the top of the syringe clamp, seen in Fig. 25A, a similar construction must be used. Instead of positioning the syringe barrel thru hole, here we need to position the syringe plunger thru hole. By placing a 100° wedge spaced 4 mm from the mounting side of the clamp a construction circle the same diameter of the syringe barrel can be drawn as in Fig. 25B. A circle concentric to this construction, with a diameter capable of passing the syringe plunger, can then be easily drawn. The slot dimension should also be adjusted to allow for disassembly of the clamp without removal of the plunger.

The last step prior to using the Replistruder 4 is to connect the motor to the control system and calibrate its motion. Earlier in this guide we discussed the theoretical steps per mm for the carriage of the Replistruder 4. With a 400 step per rotation stepper motor, a 3:1 gearing ratio, and an M8 leadscrew the result is 960 full steps per rotation, which can then be multiplied by the degree of microstepping implemented. It is advisable to calibrate the Replistruder 4 by measuring carriage travel using a pair of calipers or other suitably accurate measuring device. Begin with the theoretical steps per mm in the firmware. Next, measure the initial position of the carriage from a reference plane. Move the carriage a known distance, such as 5 cm, then measure the actual travel. This measurement can be used to proportionally alter the steps per mm to calibrate the travel of the Replistruder 4.

How the steps/mm of the extruder axis of a 3D printer can be modified depends on the printing platform. For some printers, the modification is as simple as using the M502, M92, and M500 GCode commands to alter the settings stored in the EEPROM. For others, it may be necessary to find the uncompiled firmware from the manufacturer, modify the steps/mm definitions and compile and flash the firmware to the printer. Our preferred implementation is with newer 32-bit printer boards such as the Duet3D Duet 2 WiFi. Amongst a broad range of features these boards allow for easy modification of printer parameters through a text file that is editable on a web interface hosted by the board itself. Other parameters that may be necessary to modify could be motor current (we use 80% of the motor’s rated current), as well as velocity and acceleration characteristics.

Maintenance of the Replistruder 4 during prolonged use may be necessary. It is important to check that the belt remains tightened. It is also possible that the leadscrew nut, over time, may become worn. To fix this it is a simple matter of disassembling the carriage and replacing the nut. It is possible that additional lubrication may delay this process, but it is not necessary.

## 7 Validation and characterization

**Fig. 26:**
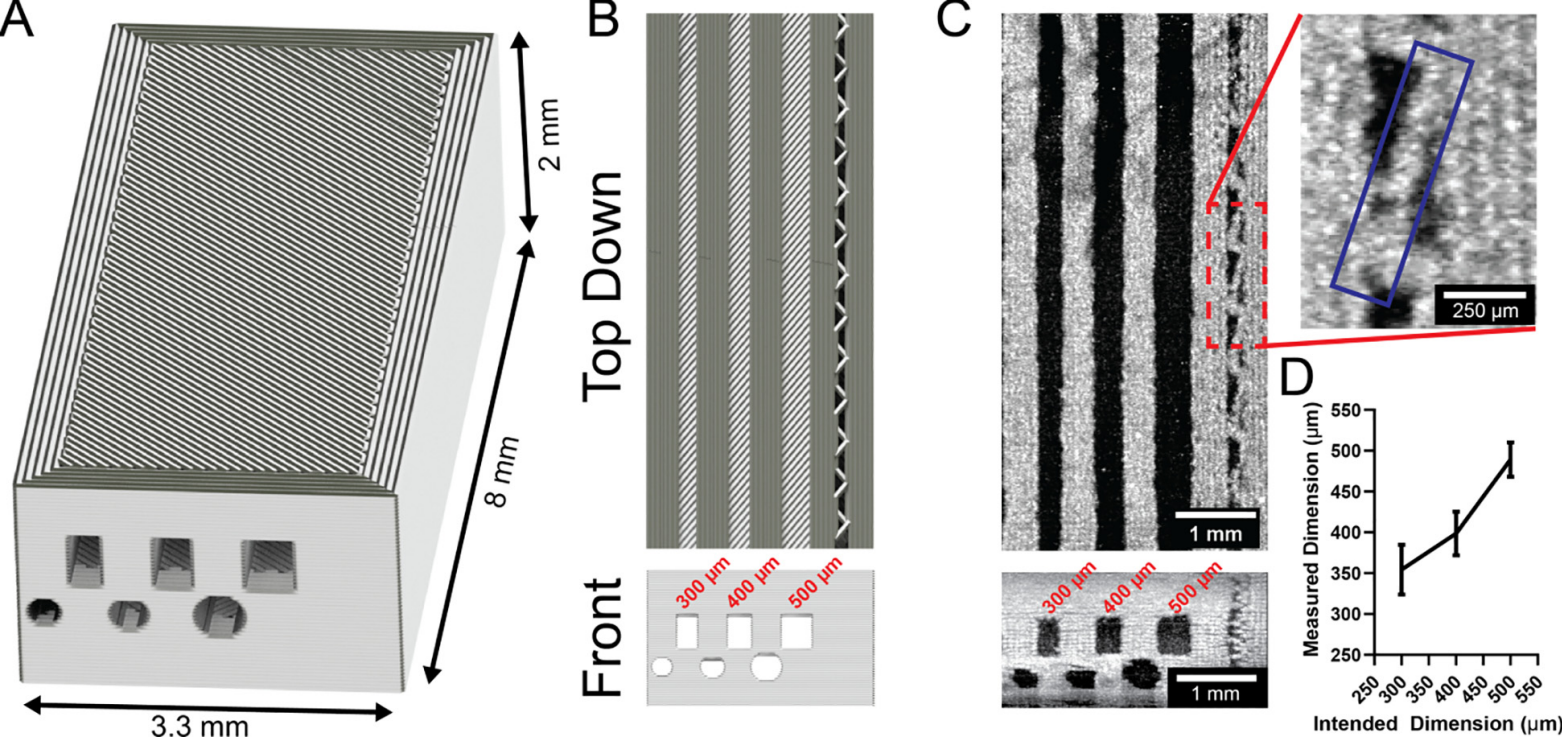
**Replistruder 4 Print Resolution Test. A)** Isometric view render of a resolution test. **B)** Top down and front view renders of the resolution print **C**) Top down and front view OCT images of three channels in a collagen resolution print. The inset calls attention to a single short line of infill in the wall of the print (blue box). **D)** We quantified the dimensions of the 300, 400, and 500 µm channels, all three channels were statistically significantly different from each other by ANOVA followed by Tukey’s p < .0001. (For interpretation of the references to colour in this figure legend, the reader is referred to the web version of this article.)

**Fig. 27:**
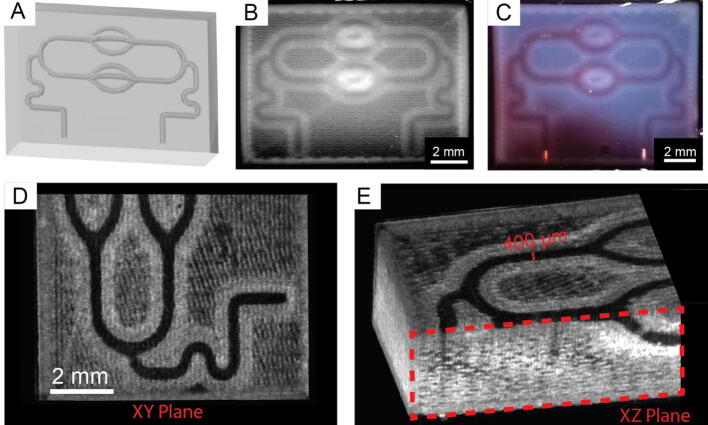
**Replistruder Functional Print. A)** An isometric view of a microfluidic network with multiple bifurcations, including out of plane branches. **B)** A view of the microfluidic network printed in collagen type I. **C)** A view of the microfluidic network after perfusing it with a red dye. **D)** A top down view of an optical coherence tomography volumetric image of the network, showing that it is fully open as printed. **E)** An isometric view of the network, showing the out of plane branches (bottom right corner, red box) were printed and patent as well. (For interpretation of the references to colour in this figure legend, the reader is referred to the web version of this article.)

To validate a syringe pump designed for extrusion-based 3D printing one of the most relevant benchmarks is the quality of printed objects. After developing the Replistruder 4 we created a resolution test design including 500, 400, and 300 µm wide channels (Fig. 26A, B) and printed it with a 100 µm needle using collagen type I with the FRESH technique. We imaged the print using optical coherence tomography (OCT), an infrared light based optical imaging technique that can be used to acquire high resolution 3D images through highly light-scattering samples (such as the 3D printed collagen constructs presented here). In Fig. 26C we can see a top down and front view of this print, in the inset we can see a zoom in of the infill. To determine the volume of a filament (approximating it as a rectangular prism) we need the length of a filament, its diameter, and its height. A line of infill is highlighted in the inset (blue box) which is 670 µm long; when printing with 50 µm layers and a 100 µm needle this represents a 3.35 nL volume. We also investigated the accuracy of the channel widths using image segmentation in Fig. 27D. The mean widths of the 500 and 400 µm wide channels were 489.1 ± 21.09 µm and 398.7 ± 26.83 µm respectively, while the 300 µm wide channel was 354.3 ± 30.68 µm, suggesting further printing optimization may be necessary. All three channels were significantly different from each other by one-way ANOVA followed by Tukey’s multiple comparison test with p < .0001 within one sample.

While assessment of test objects and filaments is relevant, for bioprinting the goal is to produce functional, biologically inspired constructs. To that end we developed a collagen microfluidic model, shown in Fig. 27A, with 400 µm wide channels. In Fig. 27B we can see the model printed in collagen type I, with the network clearly visible. We successfully perfused the network with a red dye, shown in Fig. 27C. Using OCT we can clearly see that the channels are patent in Fig. 27D. The measured channel width, taken from multiple parts of the image, was 368 ± 25.7 µm. We can see that the out of plane components of the network were also patent in the XZ cut plane view in Fig. 27E. Together these validation prints demonstrate the Replistruder 4′s high fidelity printing capabilities and have shown that precise control over extrusion and retraction directly impacts our ability to print functional, high resolution constructs. We have adopted the Replistruder 4 as our default syringe pump on our lab-built open-source printers and in our annual printer conversion workshops (Supplemental Video 1). We hope to share its capabilities with other researchers by releasing it in this publication.

Video 1
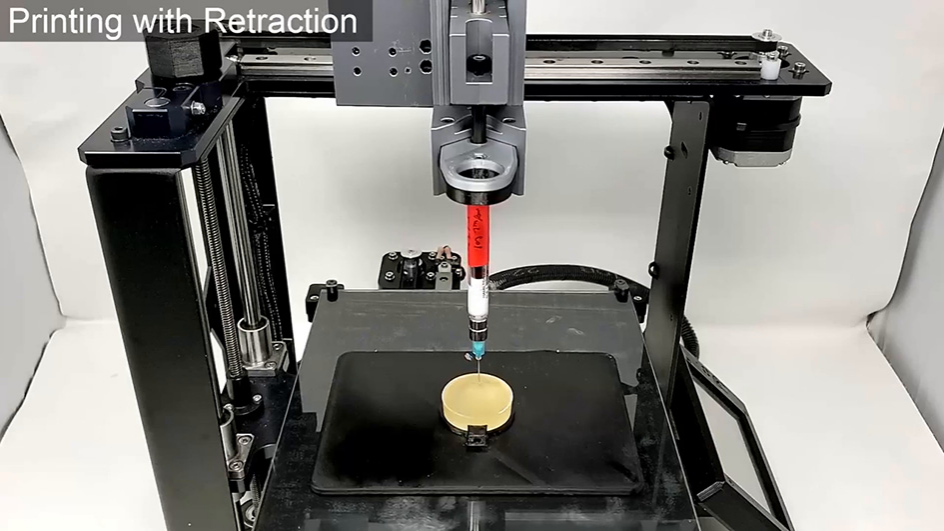

## Declaration of Competing Interest

The authors declare that they have no known competing financial interests or personal relationships that could have appeared to influence the work reported in this paper.
